# *Notes from the Field*: Measles Outbreak Associated with International Air Travel — California, March–April 2017

**DOI:** 10.15585/mmwr.mm6925a6

**Published:** 2020-06-26

**Authors:** Lihan Lu, Efrosini Roland, Eric Shearer, Matthew Zahn, Maria Djuric, Eric McDonald, Susan Redd, Kara Tardivel

**Affiliations:** ^1^Division of Global Migration and Quarantine, National Center for Emerging and Zoonotic Infectious Diseases, CDC; ^2^Eagle Medical Services, Atlanta, Georgia; ^3^Orange County Health Care Agency, California; ^4^County of San Diego Health & Human Services Agency, California; ^5^Division of Viral Diseases, National Center for Immunization and Respiratory Diseases, CDC

On March 14, 2017, the County of San Diego Health and Human Services Agency (COSD HHSA) notified CDC of a measles case in an adult airline passenger (patient A), with recent travel to Indonesia. The patient had developed rash and swollen eyes during a flight from Hong Kong to Los Angeles on March 8, followed by conjunctivitis and cough after arrival; the patient proceeded to an urgent care clinic, but a measles diagnosis was not considered. On March 9, patient A visited the clinic again, at which time measles was confirmed by polymerase chain reaction (PCR) testing on March 14. Patient A reported having received 1 dose of measles, mumps, and rubella (MMR) vaccine. CDC identified 22 contacts from the flight, involving seven U.S. states and two countries; potentially exposed flight crew were notified on March 15. COSD HHSA identified 483 community contacts, 81 of whom received self-quarantine recommendations because they lacked presumptive evidence of immunity.*

On March 28, COSD HHSA confirmed measles in patient B, an adult with unknown vaccination status, who had been exposed to patient A in the clinic waiting room during patient A’s first visit on March 8. Serologic testing indicated that patient B was not immune to measles, and the patient had been instructed to self-quarantine until March 29. On March 24, patient B developed fever, cough, and sore throat and visited an urgent care clinic, informed the clinician of the measles exposure, but measles was not considered. The patient notified COSD HHSA when rash developed on March 25; measles was confirmed March 27 by PCR testing. Contact investigation of patient B identified 31 contacts, most linked to a home-based day care center where patient B resided, resulting in self-quarantine recommendations for six persons because they lacked presumptive evidence of immunity.

On March 31, the Orange County Health Care Agency (OCHCA) notified CDC of a measles case in patient C, a flight attendant who served patient A during the March 8 flight and reported having received 2 MMR doses. Patient C developed a mild cough on March 23 while working on a flight to the United States and developed subjective fever and a rash the next day. Patient C visited an urgent care clinic on March 25 and tested negative for measles immunoglobulin M; however, specimens collected March 30 by OCHCA and tested by PCR confirmed measles. The March 23 flight contact investigation included 164 passengers from 27 states and eight countries; OCHCA identified 12 community contacts, all of whom had documentation of immunity.

On April 3 and 10, OCHCA confirmed measles in two siblings. Patients D and E, aged 14 and 12 years, developed rash on April 2 and April 11, respectively. They resided in the same county as patient C, and neither had received measles-containing vaccine. Investigation at three community exposure sites identified 338 contacts and resulted in school exclusion of six students lacking documentation of immunity, including issuance of one quarantine order. Further investigations could not establish a link between patient C and patients D and E. Isolates from all five patients (A–E) were genotyped as D8 with an identical corresponding nucleotide sequence (N450) and were the only isolates identified in the United States during March–April 2017.

This travel-associated measles outbreak serves to remind travelers, airlines, clinicians, and the public that vaccine-eligible adult travelers lacking evidence of immunity should receive 2 MMR doses before traveling internationally (*1*). Clinicians should always consider measles when evaluating patients with febrile rash illness and international travel histories and in any patient reporting measles exposure, regardless of rash. Persons with recent known exposure to measles, regardless of vaccination history, should self-isolate at the first sign of illness and immediately contact their local public health authority.

Contact investigation during measles outbreaks is costly to the public health system and labor-intensive; this investigation identified approximately 1,000 contacts who required follow-up (*2*). The high communicability of measles continues to challenge identification of epidemiologic linkage during measles investigations. International travel, particularly to countries with endemic measles or measles outbreaks, presents a risk for exposure and subsequent introduction to U.S. communities (*3*,*4*). Measles cases in flight attendants, including the case from this outbreak, prompted CDC to issue new measles recommendations for airlines (*5*).

## Footnotes

* Acceptable presumptive evidence of immunity to measles include at least one of the following: 1) written documentation of adequate vaccination (2 doses of measles-containing vaccine administered at least 28 days apart for school-aged children, adolescents, and adults at high risk, including international travelers; or 1 or more dose of measles-containing vaccine administered on or after first birthday for preschool-aged children and adults not at high risk), 2) laboratory confirmation of measles, 3) laboratory evidence of immunity, or 4) birth before 1957.
